# Ultrasensitivity without conformational spread: A mechanical origin for non-equilibrium cooperativity in the bacterial flagellar motor

**Published:** 2025-02-05

**Authors:** Henry H. Mattingly, Yuhai Tu

**Affiliations:** 1 Center for Computational Biology, Flatiron Institute, New York, NY, USA; 2 IBM Thomas J. Watson Research Center, Yorktown Heights, NY, USA

## Abstract

Flagellar motors enable bacteria to navigate their environments by switching rotation direction in response to external cues with high sensitivity. Previous work suggested that ultrasensitivity of the flagellar motor originates from conformational spread, in which subunits of the switching complex are strongly coupled to their neighbors as in an equilibrium Ising model. However, dynamic single-motor measurements indicated that rotation switching is driven out of equilibrium, and the mechanism for this dissipative driving remains unknown. Here, based on recent cryo-EM structures, we propose that local mechanical torques on motor subunits can affect their conformation dynamics. This gives rise to a tug of war between stator-associated subunits, which produces cooperative, non-equilibrium switching responses without requiring nearest-neighbor interactions. Since subunits are effectively coupled at a distance, we call this mechanism “Global Mechanical Coupling.” Our model makes a qualitatively new prediction that the motor response cooperativity grows with the number of stators driving rotation. Re-analyzing published motor dose-response curves in varying load conditions, we find tentative experimental evidence for this prediction. Finally, we show that operating out of equilibrium enables motors to achieve high cooperativity with faster responses compared to equilibrium motors. Our results suggest a general role for mechanics in sensitive chemical regulation.

The bacterial flagellar motor (BFM) is a complex, macromolecular machine [1–4] that propels motile bacteria. In response to external cues, the BFM can switch its direction of rotation, e.g. from counter-clockwise (CCW) to clockwise (CW), which reorients the cell body and enables directed navigation [5]. Different species exhibit diverse motor architectures and swimming patterns [6], yet many of the core components are conserved [7–9].

While much is known about the BFM, fundamental questions about the rotation switching decision remain. In *Escherichia coli*, switching is extremely sensitive to the internal concentration of the response regulator CheYp, with a Hill coefficient of at least 10 [10, 11]. Based on the observation that the switch complex contains a ring of ∼ 34 FliM subunits to which CheYp binds [12], Duke et al. [13] proposed a “conformational spread” mechanism wherein each FliM subunit can be in one of two conformational states (CW or CCW), and neighboring FliM’s prefer to be in the same conformation. Quantitatively, the conformational spread mechanism can be described by an equilibrium Ising-type model with strong nearest-neighbor coupling, which leads to a high Hill coefficient in agreement with experiments [13, 14].

However, measurements of single motor switching dynamics have found that the distributions of CCW and CW intervals exhibit a peak, first observed by Korobkova et al. [15] and later confirmed by Wang et al. [16] (see also [17]). Theoretically, it was shown by Tu [18] that peaked interval-time distributions cannot be generated by any equilibrium process. This general result implies that the BFM switching dynamics must be coupled to energy dissipation, mostly likely the ion motive force which drives rotation of the flagellum. Subsequent theoretical work has added non-equilibrium effects *ad hoc* to the conformational spread model [16, 21, 22], but the physical mechanism by which rotation switching is coupled to dissipation remains unclear.

Recently, key insights about the rotation switching mechanism emerged from cryo-EM structures of BFMs [23, 24]. First, the MotAB stators that drive motor rotation in many bacterial species are composed of 5 MotA and 2 MotB monomers, and they themselves rotate. Torque is transduced to the flagellar rotor through electrostatic interactions between MotA and FliG in the C-ring at the base of the flagellum. Thus, the C-ring can be thought of as a large gear that is rotated by smaller stator gears, where MotA and FliG are the cogs [25]. Structures of motors locked in different rotation states revealed that FliG subunits interact with the inner edge of the stators during CCW rotation, but change pose by 20–30 degrees to interact with the outer edge during CW rotation ([26, 27], Fig. 1A). This suggests that stators always rotate CW, and conformation changes in FliG cause the flagellum to switch rotation direction. Binding of CheYp to FliM then biases its associated FliG towards the outer conformation to induce CW rotation.

Based on these structures, we propose a model for how the internal mechanics of the motor could coordinate FliG subunits and generate a non-equilibrium, cooperative switching response without requiring conformational spread. The central idea is that, when the conformation of one FliG subunit differs from the rest, the local torque from its stator could mechanically push it into alignment with the others. Since forces transduced through the C-ring effectively couple all stator-engaged FliG subunits, we call this “Global Mechanical Coupling” (GMC). Using a coarse-grained theory and simulations, we show that GMC dissipates energy to generate high co-operativity. Furthermore, GMC uniquely predicts that the Hill coefficient of the switching response increases with the number of stators driving rotation, and we provide tentative evidence for this prediction. Finally, we show that GMC works synergistically with the nearest-neighbor couplings of conformational spread, easing a speed-sensitivity trade-off faced by purely equilibrium models. The GMC mechanism provides an unique starting point for understanding how chemical and mechanical inputs are integrated into motor function.

## RESULTS

### Local torques can drive FliG conformation cycles

**FIG. 1.**
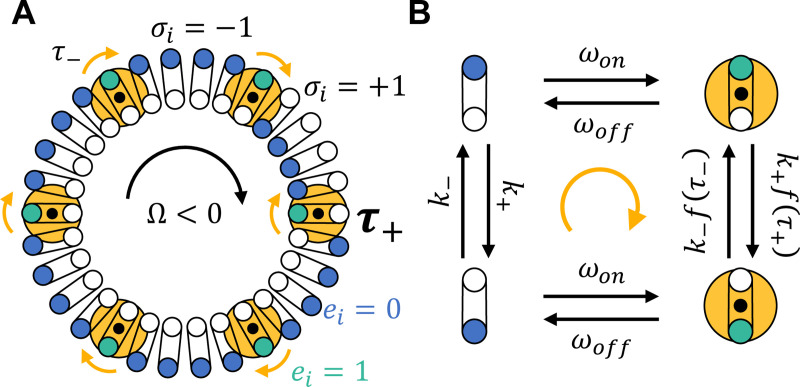
A) Schematic top view of the motor, showing FliG subunits of the C-ring (blue, green circles) and stators (yellow circles) (see e.g. [2–4, 37]). At a moment in time, FliG subunits are either in an inner conformation $\left(\sigma_{i}=+1\right)$ or outer conformation $\left(\sigma_{i}=-1\right)$, and they can be engaged with a stator (green, $e_{i}=1$) or unengaged (blue, $e_{i}=0$). FliG subunits translate through space, engaging and unengaging with stators, as the C-ring rotates at angular velocity Ω. Stators are fixed in place and exert torques in the directions of the yellow arrows. When a stator-engaged FliG subunit is unaligned with the other engaged subunits, C-ring rotation drives it against the torque of its stator. Thus, this subunit experiences a larger torque (here, $\tau_{+}$) than those aligned with the rotation direction (here, $\tau_{-}$). B) Local torques can drive FliG conformation dynamics out of equilibrium.

What happens when one FliG subunit adopts a conformation that is different from the rest? As the C-ring rotates, that subunit will encounter a stator and be pushed *against the stator’s torque* (Fig. 1A). Thus, the unaligned subunit should experience a larger torque than those aligned with the direction of rotation. If this local torque mechanically pushes the subunit towards the opposite conformation, this could coordinate subunit conformation dynamics.

To make this picture mathematical, we construct a minimal model of FliG-stator mechanics and FliG conformation dynamics. We model the C-ring as a ring of N FliG subunits, where each site is characterized by a binary conformation, σ∈{−1,+1}, and a binary stator-engagement state, e∈{0,1} (Fig. 1AB). Here, σ=+1 and −1 correspond to the inner and outer conformations of FliG subunits observed in cryo-EM experiments. Then, e=1 when a subunit is in contact with a stator, and e=0 otherwise. The number of stators driving the flagellum is $M=\sum_{i=1}^{N}e_{i}(t)$. M is known to adapt to external load on a time scale of minutes [28–35], up to M≈11 [36], but we will consider constant-load environments and take M to be constant.

Subunits that are not engaged with a stator, e=0, switch conformation from σ=+1→−1 and vice versa at rates $k_{+}$ and $k_{-}$:

(1) $$k_{\pm }=k_{0}\;\text{exp}(\mp \text{Δ}F/2).$$

The free energy difference ΔF, between conformations σ=+1 and −1 includes the effects of CheYp binding and intrinsic energy differences, and $k_{0}$ is the switching rate when ΔF=0. Throughout, we rescale energy by the scale of thermal fluctuations, $k_{B}\;T\approx 4.1$ pN nm at room temperature.

When engaged with a stator, the switching rates are modified by the torque, τ, exerted by the stator on the subunit:

(2) $$k_{+}^{e}=k_{+}f\left(\tau_{+}\right),\;k_{-}^{e}=k_{-}f\left(\tau_{-}\right)$$

where $\tau_{\pm }$ is the torque on subunits in conformation σ=±1, and f(τ) is a monotonically increasing function. Here, we take:

(3) f(τ)≡exp(γτ).

This functional form is commonly used to model slip-bonds [38, 39], where protein-protein or protein-ligand dissociation rates increase with force along a reaction coordinate. With local torque τ rescaled by the stator stall torque $\tau_{0}$ [19, 40–42], the dimensionless parameter $\gamma \approx \alpha \;\tau_{0}\text{Δ}\phi /\left(k_{B}T\right)$ includes: the projection of the stator torque along the direction of the FliG conformation transition, α; the difference in pose angles of the two FliG conformations, Δϕ≈0.4 radians [26, 27]; and the scale of thermal fluctuations. Below, we will vary the value of γ, but plugging in numbers, with α≈0.1, we get γ≈3.2.

The conformation switching dynamics of this model dissipate energy. In the mean field limit, given the states of the rest of the subunits, a single FliG subunit can be in one of four states (Fig. 1B). In addition to conformation switching dynamics, subunits transition between stator-engaged and -unengaged states with rates that depend on the motor rotation speed $\omega_{on/off}\propto \text{Ω}$. With four states, a subunit can undergo cycles in its phase space, and the free energy dissipated per cycle is:

(4) $$\text{Δ}G=\text{ln}\left(\frac{\omega_{on}k_{+}f\left(\tau_{+}\right)\omega_{off}k_{-}}{k_{+}\omega_{on}k_{-}f\left(\tau_{-}\right)\omega_{off}}\right)=\gamma \left(\tau_{-}-\tau_{+}\right),$$

using f(τ) above. Thus, detailed balance is broken as long as γ≠0 and $\tau_{-}\neq \tau_{+}$. Microscopically, $\tau_{-}\neq \tau_{+}$ because torque is distributed evenly among subunits, so those in the majority conformation experience smaller torques per subunit. Thus, rotation switching can be driven out of equilibrium by torque-dependent conformation dynamics. Rather than ATP hydrolysis, the energy source for this driving is the ion motive force across the cell membrane, which is necessary for the stators to apply torques on FliG and the C-ring.

### Minimal model of motor mechanics

Next, we need a model for the local torque on stator-engaged FliG subunits. We assume subunits have strong interactions with stators, so that the two rotate together like gears. Furthermore, we assume that the rotor rotates as a rigid object. We also assume that the system is in the over-damped limit, that stator refueling and release of ions are fast, and we neglect fluctuations in torque. Below, we rescale time by the motor’s zero-load speed, ${\text{Ω}}_{0}$, and torque by the stator stall torque, $\tau_{0}$, which in *E. coli* are ${\text{Ω}}_{0}\approx 300\;\text{Hz}$ and $\tau_{0}\approx 300$ pN nm [19, 40–42].

With these assumptions, we write down torque balances on each stator-engaged FliG subunit and on the rotor, along with no-slip conditions between FliG’s and stators (SI). Solving these equations for the dimensionless angular velocity of the motor, Ω(t), gives:

(5) $$\text{Ω}(t)=\frac{M/\beta }{1+M/\beta }\left(2\frac{N_{e}^{+}(t)}{M}-1\right),$$

where β is the dimensionless rotational drag on the flagellum and $N_{e}^{+}(t)=\sum_{i|\sigma_{i}(t)=+1}e_{i}(t)$ is the number of stator-engaged FliG subunits in the σ=+1 conformation. Thus, the rotation velocity depends linearly on the number of engaged subunits in the +1 conformation, and the rotation direction is set by the majority conformation among engaged subunits. At full alignment ($N_{e}^{+}=0$ or $N_{e}^{+}=M$), the rotation speed depends M/β, which can be understood as the ratio of the motor’s output stall torque relative to the drag torque at the zero-load rotation speed (SI). From recent experiments in *E. coli* [43], M≈4−6 and β≈2.5 during swimming.

The torque balances also give us the magnitude of the local torque on stator-engaged FliG subunits:

(6) $$\tau_{\pm }(t)=1\mp \text{Ω}(t).$$

As previewed earlier, $\tau_{-}\neq \tau_{+}$ when the motor is rotating. Together with Eqn. (3), the expression in Eqn. (6) implies that local torques change both the free energy difference and the energetic barrier between conformations σ=+1 and −1.

Since Ω(t) depends on the states of *all* stator-engaged subunits, local torques on subunits depend on the global state of the motor. Eqns. (5) and (6) indicate that FliG subunits that are in the majority conformation experience smaller torques than those in the minority, and the difference between them increases as the size of the majority increases. Furthermore, the local torque on stators in the minority exceeds their stall torque $\left(\tau_{\pm }>1\right)$. With the no-slip condition, this predicts that stators in the minority can be overpowered by the majority and forced to rotate CCW, *against* their natural rotation direction (SI Fig. 1).

### Global mechanical coupling coordinates subunits

The switching rates and local torques above indicate that the stator-engaged subunits are in a tug of war. Say the motor is initially not rotating, Ω(t)=0, and therefore half of the engaged subunits are in the conformation σ=+1, $N_{e}^{+}=M/2$. When one subunit flips conformation by chance, the tie is broken and the motor begins to rotate. Then, subunits in the minority conformation experience a larger torque than those in the majority (Eqn. (6)), increasing the rate at which they flip into the majority (Eqns. (2) and (3)). This creates a positive feedback loop that rapidly pushes all subunits into alignment.

To study this model quantitatively, we first considered a coarse-grained theory. Motivated by the fact that the motor rotation velocity and local torques only depend on $N_{e}^{+}$, we consider the dynamics of just two variables: the number of engaged, $N_{e}^{+}(t)$, and unengaged, $N_{u}^{+}(t)$, FliG subunits in the σ=+1 conformation. Conservation of stators determines $N_{e}^{-}(t)$, $N_{e}^{+}(t)+N_{e}^{-}(t)=M$, and conservation of FliG subunits determines $N_{u}^{-}(t)$, $N_{u}^{+}(t)+N_{u}^{-}(t)=N-M$ where $N_{e}^{-}$ and $N_{u}^{-}$ the number of engaged or unengaged subunits in the σ=−1 conformation. While this coarse-graining fully captures the conformation switching dynamics, exchange of stator-engaged FliG subunits depends on the full ring structure and must be approximated (SI). To reduce the number of model parameters, we will take M/β≫1.

In the coarse-grained model, we can derive the stationary distribution of $N_{e}^{+}$, and thus Ω. When $k_{0}\gg 1$, we find:

(7) $$P\left(N_{e}^{+}\right)=\frac{1}{Z}\left(\begin{array}{c} M\\ N_{e}^{+} \end{array}\right)\text{exp}\left(\text{Δ}FN_{e}^{+}+2\gamma M{\left(\frac{N_{e}^{+}}{M}-\frac{1}{2}\right)}^{2}\right)$$

with normalization constant Z. The stationary distribution $P\left(N_{e}^{+}\right)$, like any one-dimensional projection, has the form of a Boltzmann distribution [44]. The binomial prefactor is an entropic term that favors a monomodal distribution centered on $N_{e}^{+}=M/2$, the no-rotation state. In the exponential is an effective Hamiltonian that has contributions from the bias, ΔF, and a term of non-equilibrium origin that is equivalent to a sum of effective energetic couplings among all pairs of stator-engaged subunits (SI). The latter, positive-feedback term favors macrostates with consensus among engaged subunits, $N_{e}^{+}=0$ or $N_{e}^{+}=M$. As γ increases, this term leads to a bimodal distribution of $N_{e}^{+}$. In the limit of large γ, eventually all weight is on the two consensus macrostates, resulting in a non-equilibrium Monod-Wyman-Changeux (MWC) model [45] for motor rotation (SI Eqn. 38). Thus, the internal mechanics effectively couple all stator-engaged FliG subunits, hence the name “Global Mechanical Coupling” (GMC).

**FIG. 2.**
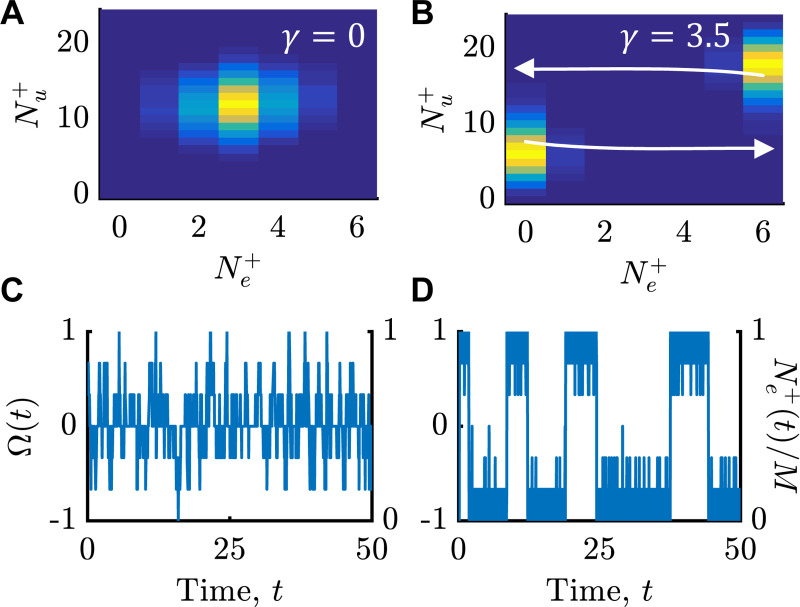
Microscopic Gillespie simulations of the full FliG ring, projected into the subspace $\left(N_{e}^{+},N_{u}+\right)$. Throughout, we use N=30, $k_{0}=1$, M=6, M/β≫1, and ΔF=0, unless specified otherwise. A,B) Stationary distributions of $\left(N_{e}^{+},N_{u}+\right)$, with probability increasing from dark to light. A) Independent switching of FliG subunits (γ=0). B) Increasing γ leads to a bimodal distribution. White arrows show average transition paths from state $N_{e}^{+}=0$ to $N_{e}^{+}=M$, and vice versa. C,D) Time series of motor rotation velocity Ω or $N_{e}^{+}/M$ (Eqn. (5)). C) When γ=0 (as in A), rotation velocity fluctuates around zero. D) At larger γ (as in B), the motor shows extended bouts of rotation at nearly full speed in either direction.

Gillespie simulations [46] of the “microscopic” ring structure, projected onto the subspace $\left(N_{e}^{+},N_{u}^{+}\right)$, agreed with these predictions and provided motor switching dynamics (Methods, Fig. 2). When there was no mechanical coupling, γ=0, the time series of motor state showed binomial fluctuations around $N_{e}^{+}=M/2$ (Fig. 2AC). At higher values of γ, the stationary distribution of $\left(N_{e}^{+},N_{u}^{+}\right)$ became bimodal (Fig. 2B). Furthermore, the time series of motor state showed extended bouts of rotation at nearly full speed in either direction, Ω(t)≈±1, with fast transitions (Fig. 2D), similar to experimental measurements (e.g. [14]). Furthermore, switching of rotation direction from CW to CCW and vice versa took different transition paths (Fig. 2B; SI; [47, 48]), a clear indication of broken detailed balance.

### GMC produces high response cooperativity at the cost of free energy dissipation

A striking feature of the BFM is the high cooperativity of its responses to intracellular CheYp concentration. To quantify cooperativity in the GMC model, we computed the Hill coefficient, H, of the stationary mean $\langle N_{e}^{+}\rangle /M$ (and thus of rotation velocity 〈Ω〉) as a function of the free energy bias ΔF [13, 49].

In the coarse-grained theory:

(8) $$H\approx \frac{4}{M}\partial_{\text{Δ}F}\langle N_{e}^{+}\rangle {|}_{\text{Δ}F=0}\approx \frac{4}{M}Var\left(N_{e}^{+}\right){|}_{\text{Δ}F=0}\leq M.$$

**FIG. 3.**
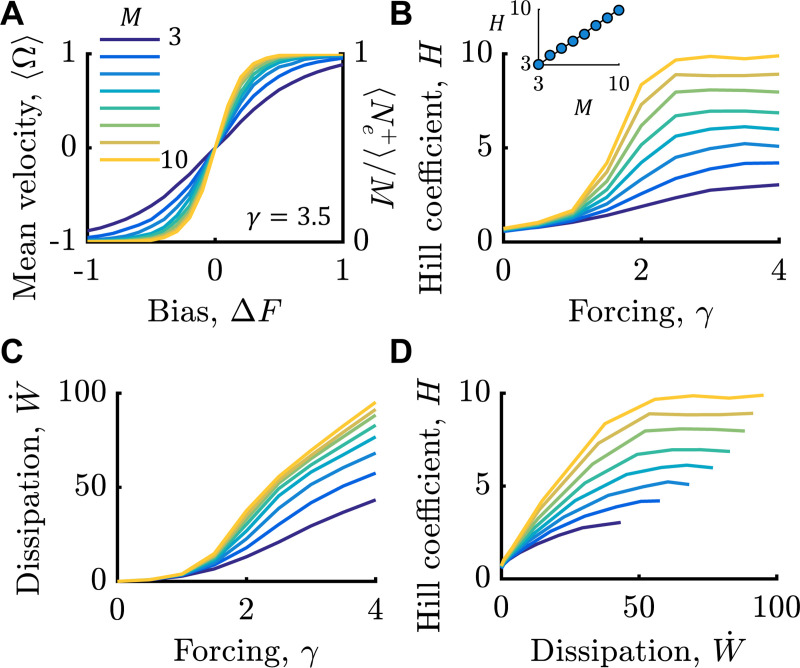
Non-equilibrium motor cooperativity. A) Stationary mean rotation velocity, 〈Ω〉, or $\langle N_{e}^{+}\rangle /M$, versus free energy bias, ΔF. Responses for varying M are shown, increasing from 3 (light) to 10 (dark), with γ=3.5 fixed. Colors are the same in all panels. B) Hill coefficients H as a function of γ, for various values of M. Inset is H versus M at γ=4, where markers are from simulation data and the solid line is y=x. C) Free energy dissipation rate versus γ, for various values of M. D) Hill coefficient (B) versus dissipation rate (C).

This predicts that the Hill coefficient is proportional to the variance of $N_{e}^{+}$, which increases with γ as the distribution becomes bimodal. In the large γ limit, the Hill coefficient approaches M, the number of stators. Thus, GMC makes a qualitatively new prediction: the cooperativity of the motor switch to *chemical* inputs increases with the number of *torque generators* driving rotation. Microscopic Gillespie simulations were consistent with this prediction (Fig. 3AB). In SI Fig. 2, we verify that this prediction persists even when there is nonzero nearest-neighbor coupling between subunits, and that it requires mechanical coupling γ>0.

While increasing γ increased the cooperativity, H, it also increased the free energy dissipation rate. We computed the energy cost of cooperativity, $\dot{W}$, from the Kullback-Leibler divergence between forward and backward simulation trajectories as a function γ (Fig. 3C; Methods; [50–52]). Plotting $\dot{W}$ versus H in Fig. 3D showed that increasing dissipation “buys” steeper responses, up to the point where H saturates at M.

Recent experiments provide tentative evidence in support of the prediction that the Hill coefficient increases with the number of stators driving rotation. Zhu et al. [22] measured the CW bias of individual motors in multiple load conditions by varying the concentration of Ficoll in the experimental medium. Since stator number M increases with load due to adaptation [29, 30, 32, 34, 35], a corresponding increase in the Hill coefficient would predict specific changes in motor CW bias, assuming the average CheYp concentration in each cell is unchanged.

In particular, we used measurements of CW bias from 40 motors monitored by 0.35-*μ*m beads in 0% and 19% w/v Ficoll (Fig. 3 of [22]). In the former condition, about half of the stator sites are occupied (M≈6), while in the latter nearly all are occupied (M≈11) [22, 30]. We fit these data to estimate each cell’s internal CheYp concentration, as well as the values of the load-dependent Hill coefficient, H, and half-maximum CheYp concentration, K (details in the SI). To uniquely specify parameter values, we fixed H(19%)=10.3 and K(19%)=3.1μM, as in Ref. [22].

**FIG. 4.**
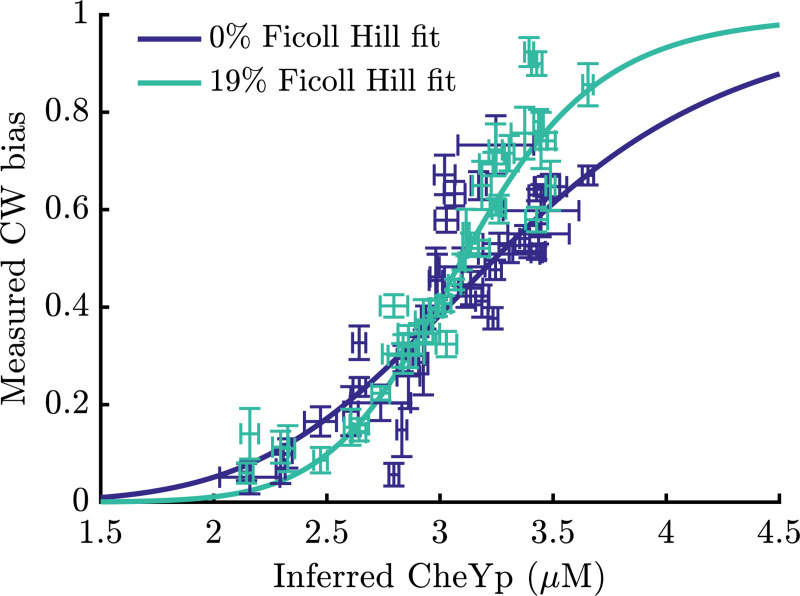
Tentative evidence for GMC. Measured CW bias versus inferred CheYp concentration for individual motors in two load conditions, 0% Ficoll and 19% Ficoll, along with Hill function fits. Error bars are standard errors, where vertical bars are measurement uncertainties and horizontal bars are uncertainties from the inference procedure. Data are from Ref. [22].

Fig. 4 shows the measured CW biases as a function of inferred CheYp concentration, along with the Hill function fits. The best-fit parameter values were H(0%)=6.0±0.3 and K(0%)=3.24±0.01μM, suggesting that the Hill coefficient nearly doubles upon an increase in load from 0% to 19% Ficoll. Since previous measurements suggest that the adapted M roughly doubles across these loads [30], and GMC predicts H∝M under certain conditions (Eqn. (8)), these results are very suggestive. Simultaneous measurements of M, CheYp, and CW bias are needed to fully test this prediction.

### GMC eases a speed-sensitivity trade-off in equilibrium models

Conformational spread can reproduce the motor’s highly cooperative response to chemical inputs without dissipating energy [13, 14]. Furthermore, while the Hill coefficient H of GMC alone is bounded by the number of stators when $k_{0}\gg 1(H\leq M)$, nearest-neighbor interactions can achieve H up to the size of the entire C-ring (H≤N) [53]. Why might the BFM dissipate energy in the switching response? Detailed balance constrains the dynamics of equilibrium models, and past work has shown that energy dissipation can generally ease tradeoffs between speed and sensitivity or accuracy [48, 54–56].

To test this possibility, we computed the Hill coefficient and the response speed, $\tau_{R}^{-1}$, for varying values of γ and nearest-neighbor coupling, J. Response speed was defined as the average time it took a motor in the stationary state with ΔF=1/2 to reach $N_{e}^{+}=0$ after a rapid change in bias to ΔF=−1/2, mimicking a tumble after a change in swimming direction. This response speed depends on the values of ΔF before and after the “stimulus,” but we expect similar qualitative behavior regardless of the specific values.

**FIG. 5.**
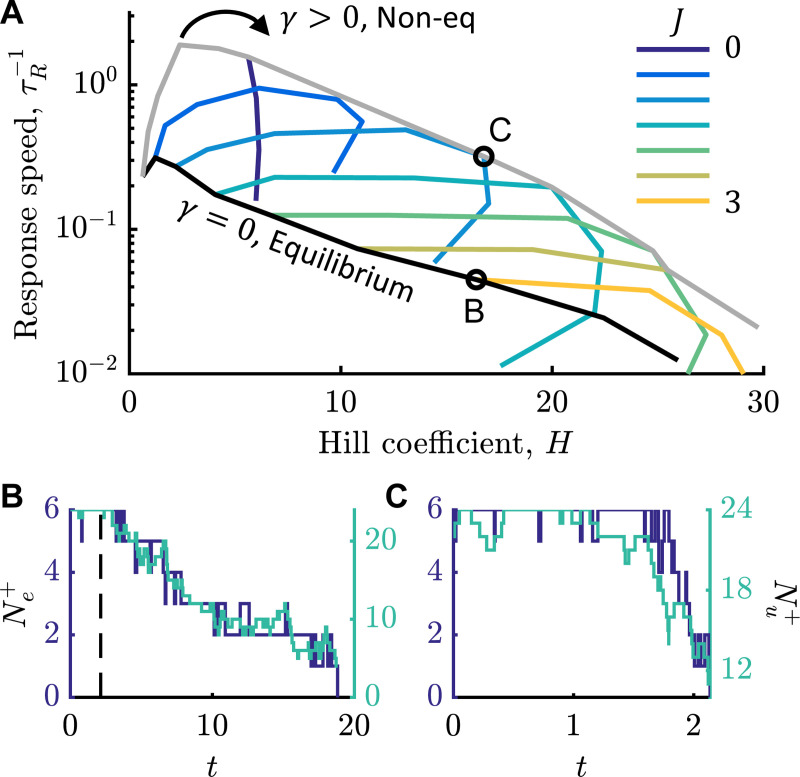
GMC eases speed-sensitivity trade-off of equilibrium motors. A) Response speed, $\tau_{R}^{-1}$, versus Hill coefficient, H, for equilibrium motors with nearest-neighbor interactions (black line, γ=0, J>0) and for non-equilibrium motors (colorful lines, γ>0, J≥0). In the latter, each color is a fixed value of J with γ increasing clockwise around the curve. Gray is the envelope of optimal strategies. B,C) Representative response trajectories of equilibrium (B) and non-equilibrium (C) motors marked in (A), which have similar H but 10-fold different $\tau_{R}^{-1}$. Plotted are time series of $N_{e}^{+}$ (blue, left axis) and $N_{u}^{+}$ (teal, right axis). Vertical dashed line in (B) is the duration of (C). In (B), J=3, γ=0. In (C), J=1, γ=2.

As J increased, equilibrium conformational spread models (γ=0) showed decreasing response speed with increasing Hill coefficient (Fig. 5A, black line). Making γ non-zero introduced the non-equilibrium effects of GMC. Initially, γ increased *both* H and $\tau_{R}^{-1}$, up to a point where H peaked and the dynamics slowed down dramatically (Fig. 5A, colorful lines). Non-equilibrium motors with both J and γ non-zero consistently achieved the same Hill coefficient as equilibrium ones with about 10 times faster responses.

Representative trajectories demonstrate why GMC enables faster responses. In Fig. 5BC, we compare an equilibrium motor and a non-equilibrium one, both of which have a Hill coefficient of H≈16. The equilibrium motor transitions as described before [13], where a ring of all σ=+1 subunits nucleates two energetically-unfavorable interfaces with a domain of σ=−1, which then grows slowly via a drift-diffusion process. Since stator engage ment is nearly independent of conformation dynamics, $N_{e}^{+}$ and $N_{u}^{+}$ change in concert. In the motor with GMC, nearest-neighbor interactions are weaker, so the weakly-coupled, unengaged spins first flip σ=−1, causing $N_{e}^{+}$ to decrease when those subunits engage with stators. Once $N_{e}^{+}$ crosses below M/2, positive feedback rapidly pushes $N_{e}^{+}$ to zero.

## DISCUSSION

Here, we proposed a biophysical mechanism for the non-equilibrium rotation switching dynamics of the bacterial flagellar motor. In this mechanism, the conformation dynamics of FliG subunits in the C-ring depend on the local torque they experience, which in turn depends on the conformations of all other subunits through rigidity of the C-ring. Since internal mechanics of the motor coordinate subunits at a distance, we call this mechanism “Global Mechanical Coupling.”

Past works have considered torque-dependent FliG conformation dynamics [16, 19–22]. One line of work treated the motor *output* torque in a given load condition as a fixed parameter that modulated the conformation dynamics of all FliG subunits [16, 19, 21, 22]. It is unclear how this phenomenological description would be implemented at the molecular level. A separate study considered the effects of instantaneous torque on individual, stator-engaged FliG subunits’ conformation dynamics [20]. However, it was again assumed that motor output torques influence FliG switching. No previous model of rotation switching considered the equal and opposite torque from the C-ring on the stators, which is required for physical consistency. Here, a torque balance on the stators and C-ring revealed that local torques on FliG subunits depend on the global motor state.

GMC makes a testable prediction that the Hill coefficient of the motor switching response to chemical inputs, H, increases with the number of stators driving rotation, M. Our analysis of experimental data from Zhu et al. [22] support this prediction, but more direct measurements are needed. If this prediction is correct, it would have major implications for current models of bacterial chemotaxis. In particular, the steep motor CW bias response curves measured by Cluzel et al. [10] and Yuan et al. [11] used motors attached to 0.5-*μ*m and 1-*μ*m beads, respectively. These are relatively large loads, leading to higher stator numbers and potentially higher Hill coefficients than cells swimming in aqueous media [43].

Although GMC can generate high cooperativity without conformational spread (nearest-neighbor interactions), which was essential in previous models, real motors may use a combination of both. FliG subunits in the C-ring are connected by a spring-shaped coil of FliM-FliN repeats [25, 26, 57], and having neighboring FliG’s in opposing conformations may induce energetically-unfavorable bending in this spring. High-resolution structures of the C-ring also suggest that steric interactions may favor alignment of neighboring FliG subunits [25, 57]. Here, we showed that nearest-neighbor interactions and GMC are synergistic: the former increases the maximum cooperativity [53], while the latter enables faster responses to inputs.

Several features of the BFM rotation switch were not included here. We lumped the effects of CheYp binding into the free energy bias ΔF, but binding has its own dynamics. Including effects of ion motive force would allow direct comparisons with more experiments. The output torque-speed curves of *E. coli* motors rotating CW versus CCW have different shapes [58], which could affect local torques on FliG subunits. More detailed modeling [59–64] could relax some of our assumptions, such as strong MotA-FliG interactions and C-ring rigidity.

The BFM is also adaptive: it adapts the number of FliM binding sites for CheYp, depending on rotation direction [65–69], and it adapts the number of stators driving rotation, depending on the external load [28–35]. GMC is a starting point for understanding how chemical and mechanical inputs are integrated into a functional switch response across a range of intracellular and environmental conditions [70].

Our results raise the possibility that dissipative mechanical forces may be leveraged for more-subtly dissipative responses to inputs in other biological contexts. A potential example is bidirectional cargo transport along microtubules [71–73]. Ongoing work [73–78] suggests opposing motors attached to the same cargo undergo a tug of war that, together with force-dependent detachment rates from microtubules, could make transport highly sensitive to chemical regulation.

## References

[1] Armitage J. P. and Berry R. M., Assembly and Dynamics of the Bacterial Flagellum, Annual Review of Microbiology 74, 181 (2020). 10.1146/annurev-micro-090816-093411 32603624

[2] Wadhwa N. and Berg H. C., Bacterial motility: machinery and mechanisms, Nature Reviews Microbiology , 1 (2021). 10.1038/s41579-021-00626-4 34548639

[3] Guo S. and Liu J., The Bacterial Flagellar Motor: Insights Into Torque Generation, Rotational Switching, and Mechanosensing, Frontiers in Microbiology 13 (2022). 10.3389/fmicb.2022.911114 PMC9195833 35711788

[4] Hu H., Santiveri M., Wadhwa N., Berg H. C., Erhardt M., and Taylor N. M. I., Structural basis of torque generation in the bi-directional bacterial flagellar motor, Trends in Biochemical Sciences Special Issue: Pushing boundaries of cryo-EM, 47, 160 (2022). 10.1016/j.tibs.2021.06.005 34294545

[5] Berg H. C. and Brown D. A., Chemotaxis in Escherichia coli analysed by Three-dimensional Tracking, Nature 239, 500 (1972). 4563019 10.1038/239500a0

[6] Grognot M. and Taute K. M., More than propellers: how flagella shape bacterial motility behaviors, Current Opinion in Microbiology 61, 73 (2021). 33845324 10.1016/j.mib.2021.02.005

[7] Chen S., Beeby M., Murphy G. E., Leadbetter J. R., Hendrixson D. R., Briegel A., Li Z., Shi J., Tocheva E. I., Müller A., Dobro M. J., and Jensen G. J., Structural diversity of bacterial flagellar motors, The EMBO Journal 30, 2972 (2011). 21673657 10.1038/emboj.2011.186 PMC3160247

[8] Zhao X., Norris S. J., and Liu J., Molecular Architecture of the Bacterial Flagellar Motor in Cells, Biochemistry 53, 4323 (2014). 24697492 10.1021/bi500059y PMC4221660

[9] Carroll B. L. and Liu J., Structural Conservation and Adaptation of the Bacterial Flagella Motor, Biomolecules 10, 1492 (2020). 33138111 10.3390/biom10111492 PMC7693769

[10] Cluzel P., Surette M., and Leibler S., An Ultrasensitive Bacterial Motor Revealed by Monitoring Signaling Proteins in Single Cells, Science 287, 1652 (2000). 10698740 10.1126/science.287.5458.1652

[11] Yuan J. and Berg H. C., Ultrasensitivity of an Adaptive Bacterial Motor, Journal of Molecular Biology 425, 1760 (2013). 23454041 10.1016/j.jmb.2013.02.016 PMC3830563

[12] Thomas D. R., Morgan D. G., and DeRosier D. J., Rotational symmetry of the C ring and a mechanism for the flagellar rotary motor, Proceedings of the National Academy of Sciences 96, 10134 (1999). 10.1073/pnas.96.18.10134 PMC17855 10468575

[13] Duke T. A. J., Le Novère N., and Bray D., Conformational spread in a ring of proteins: a stochastic approach to allostery, Journal of Molecular Biology 308, 541 (2001). 11327786 10.1006/jmbi.2001.4610

[14] Bai F., Branch R. W., Nicolau D. V., Pilizota T., Steel B. C., Maini P. K., and Berry R. M., Conformational Spread as a Mechanism for Cooperativity in the Bacterial Flagellar Switch, Science 327, 685 (2010). 20133571 10.1126/science.1182105

[15] Korobkova E. A., Emonet T., Park H., and Cluzel P., Hidden Stochastic Nature of a Single Bacterial Motor, Physical Review Letters 96, 058105 (2006). 16486999 10.1103/PhysRevLett.96.058105

[16] Wang F., Shi H., He R., Wang R., Zhang R., and Yuan J., Non-equilibrium effect in the allosteric regulation of the bacterial flagellar switch, Nature Physics 13, 710 (2017).

[17] Tu Y., Driven to peak, Nature Physics 13, 631 (2017).

[18] Tu Y., The nonequilibrium mechanism for ultrasensitivity in a biological switch: Sensing by Maxwell’s demons, Proceedings of the National Academy of Sciences 105, 11737 (2008). 10.1073/pnas.0804641105 PMC2575293 18687900

[19] Yuan J., Fahrner K. A., and Berg H. C., Switching of the Bacterial Flagellar Motor Near Zero Load, Journal of Molecular Biology 390, 394 (2009). 19467245 10.1016/j.jmb.2009.05.039 PMC2742947

[20] Bai F., Minamino T., Wu Z., Namba K., and Xing J., Coupling between Switching Regulation and Torque Generation in Bacterial Flagellar Motor, Physical Review Letters 108, 178105 (2012). 22680910 10.1103/PhysRevLett.108.178105 PMC3558881

[21] Wang B., Niu Y., Zhang R., and Yuan J., Dynamics of Switching at Stall Reveals Nonequilibrium Mechanism in the Allosteric Regulation of the Bacterial Flagellar Switch, Physical Review Letters 127, 268101 (2021). 35029477 10.1103/PhysRevLett.127.268101

[22] Zhu S., He R., Zhang R., and Yuan J., Mechanosensitive dose response of the bacterial flagellar motor, Physical Review E 110, 054402 (2024). 39690685 10.1103/PhysRevE.110.054402

[23] Deme J. C., Johnson S., Vickery O., Aron A., Monkhouse H., Griffiths T., James R. H., Berks B. C., Coulton J. W., Stansfeld P. J., and Lea S. M., Structures of the stator complex that drives rotation of the bacterial flagellum, Nature Microbiology 5, 1553 (2020). 10.1038/s41564-020-0788-8 PMC7610383 32929189

[24] Santiveri M., Roa-Eguiara A., Kühne C., Wadhwa N., Hu H., Berg H. C., Erhardt M., and Taylor N. M. I., Structure and Function of Stator Units of the Bacterial Flagellar Motor, Cell 183, 244 (2020). 32931735 10.1016/j.cell.2020.08.016

[25] Johnson S., Deme J. C., Furlong E. J., Caesar J. J. E., Chevance F. F. V., Hughes K. T., and Lea S. M., Structural basis of directional switching by the bacterial flagellum, Nature Microbiology 9, 1282 (2024). 10.1038/s41564-024-01630-z 38459206

[26] Chang Y., Zhang K., Carroll B. L., Zhao X., Charon N. W., Norris S. J., Motaleb M. A., Li C., and Liu J., Molecular mechanism for rotational switching of the bacterial flagellar motor, Nature Structural & Molecular Biology 27, 1041 (2020). 10.1038/s41594-020-0497-2 PMC8129871 32895555

[27] Carroll B. L., Nishikino T., Guo W., Zhu S., Kojima S., Homma M., and Liu J., The flagellar motor of Vibrio alginolyticus undergoes major structural remodeling during rotational switching, eLife 9, e61446 (2020). 32893817 10.7554/eLife.61446 PMC7505661

[28] Blair D. F. and Berg H. C., Restoration of Torque in Defective Flagellar Motors, Science 242, 1678 (1988). 2849208 10.1126/science.2849208

[29] Lele P. P., Hosu B. G., and Berg H. C., Dynamics of mechanosensing in the bacterial flagellar motor, Proceedings of the National Academy of Sciences 110, 11839 (2013). 10.1073/pnas.1305885110 PMC3718179 23818629

[30] Tipping M. J., Delalez N. J., Lim R., Berry R. M., and Armitage J. P., Load-Dependent Assembly of the Bacterial Flagellar Motor, mBio 4, 10.1128/mbio.00551 (2013). PMC3747592 23963182

[31] Tusk S. E., Delalez N. J., and Berry R. M., Subunit Exchange in Protein Complexes, Journal of Molecular Biology Plasticity of Multi-Protein Complexes, 430, 4557 (2018). 10.1016/j.jmb.2018.06.039 29959924

[32] Wadhwa N., Phillips R., and Berg H. C., Torque-dependent remodeling of the bacterial flagellar motor, Proceedings of the National Academy of Sciences 116, 11764 (2019). 10.1073/pnas.1904577116 PMC6576217 31142644

[33] Nirody J. A., Nord A. L., and Berry R. M., Load-dependent adaptation near zero load in the bacterial flagellar motor, Journal of The Royal Society Interface 16, 20190300 (2019). 31575345 10.1098/rsif.2019.0300 PMC6833329

[34] Wadhwa N., Tu Y., and Berg H. C., Mechanosensitive remodeling of the bacterial flagellar motor is independent of direction of rotation, Proceedings of the National Academy of Sciences 118, e2024608118 (2021). 10.1073/pnas.2024608118 PMC8054018 33876769

[35] Wadhwa N., Sassi A., Berg H. C., and Tu Y., A multistate dynamic process confers mechano-adaptation to a biological nanomachine, Nature Communications 13, 5327 (2022). 10.1038/s41467-022-33075-5 PMC9464220 36088344

[36] Reid S. W., Leake M. C., Chandler J. H., Lo C.-J., Armitage J. P., and Berry R. M., The maximum number of torque-generating units in the flagellar motor of Escherichia coli is at least 11, Proceedings of the National Academy of Sciences 103, 8066 (2006). 10.1073/pnas.0509932103 PMC1472430 16698936

[37] Chang Y., Carroll B. L., and Liu J., Structural basis of bacterial flagellar motor rotation and switching, Trends in Microbiology 29, 1024 (2021). 33865677 10.1016/j.tim.2021.03.009 PMC8510993

[38] Bell G. I., Models for the Specific Adhesion of Cells to Cells, Science 200, 618 (1978). 347575 10.1126/science.347575

[39] Wiita A. P., Ainavarapu S. R. K., Huang H. H., and Fernandez J. M., Force-dependent chemical kinetics of disulfide bond reduction observed with single-molecule techniques, Proceedings of the National Academy of Sciences 103, 7222 (2006). 10.1073/pnas.0511035103 PMC1464324 16645035

[40] Ryu W. S., Berry R. M., and Berg H. C., Torque-generating units of the flagellar motor of Escherichia coli have a high duty ratio, Nature 403, 444 (2000). 10667798 10.1038/35000233

[41] Yuan J. and Berg H. C., Resurrection of the flagellar rotary motor near zero load, Proceedings of the National Academy of Sciences 105, 1182 (2008). 10.1073/pnas.0711539105 PMC2234112 18202173

[42] Nakamura S., Kami-ike N., Yokota J.-i. P., Kudo S., Minamino T., and Namba K., Effect of Intracellular pH on the Torque–Speed Relationship of Bacterial Proton-Driven Flagellar Motor, Journal of Molecular Biology 386, 332 (2009). 19133273 10.1016/j.jmb.2008.12.034

[43] Niu Y., Zhang R., and Yuan J., Flagellar motors of swimming bacteria contain an incomplete set of stator units to ensure robust motility, Science Advances 9, eadi6724 (2023). 37922360 10.1126/sciadv.adi6724 PMC10624342

[44] Gardiner C., Stochastic Methods: A Handbook for the Natural and Social Sciences, 4th ed. (Springer, Berlin, Heidelberg, 2009).

[45] Monod J., Wyman J., and Changeux J.-P., On the nature of allosteric transitions: A plausible model, Journal of Molecular Biology 12, 88 (1965). 14343300 10.1016/s0022-2836(65)80285-6

[46] Gillespie D. T., Stochastic Simulation of Chemical Kinetics, Annual Review of Physical Chemistry 58, 35 (2007). 10.1146/annurev.physchem.58.032806.104637 17037977

[47] Zakine R. and Vanden-Eijnden E., Minimum-Action Method for Nonequilibrium Phase Transitions, Physical Review X 13, 041044 (2023).

[48] Hathcock D., Yu Q., and Tu Y., Time-reversal symmetry breaking in the chemosensory array reveals a general mechanism for dissipation-enhanced cooperative sensing, Nature Communications 15, 8892 (2024). 10.1038/s41467-024-52799-0 PMC11480488 39406715

[49] Hill A. V., The possible effects of the aggregation of the molecules of haemoglobin on its dissociation curves, J Physiol 40, 4 (1910).

[50] Kullback S. and Leibler R. A., On Information and Sufficiency, The Annals of Mathematical Statistics 22, 79 (1951).

[51] Kawai R., Parrondo J. M. R., and den Broeck C. V., Dissipation: The Phase-Space Perspective, Physical Review Letters 98, 080602 (2007). 17359081 10.1103/PhysRevLett.98.080602

[52] Yu Q. and Tu Y., Energy Cost for Flocking of Active Spins: The Cusped Dissipation Maximum at the Flocking Transition, Physical Review Letters 129, 278001 (2022). 36638284 10.1103/PhysRevLett.129.278001 PMC10317207

[53] Owen J. A. and Horowitz J. M., Size limits the sensitivity of kinetic schemes, Nature Communications 14, 1280 (2023). 10.1038/s41467-023-36705-8 PMC9995461 36890153

[54] Lan G., Sartori P., Neumann S., Sourjik V., and Tu Y., The energy–speed–accuracy trade-off in sensory adaptation, Nature Physics 8, 422 (2012). 22737175 10.1038/nphys2276 PMC3378065

[55] Sartori P. and Tu Y., Free Energy Cost of Reducing Noise while Maintaining a High Sensitivity, Physical Review Letters 115, 118102 (2015). 26406857 10.1103/PhysRevLett.115.118102 PMC4955832

[56] Fei C., Cao Y., Ouyang Q., and Tu Y., Design principles for enhancing phase sensitivity and suppressing phase fluctuations simultaneously in biochemical oscillatory systems, Nature Communications 9, 1434 (2018). 10.1038/s41467-018-03826-4 PMC5897384 29651016

[57] Singh P. K., Sharma P., Afanzar O., Goldfarb M. H., Maklashina E., Eisenbach M., Cecchini G., and Iverson T. M., CryoEM structures reveal how the bacterial flagellum rotates and switches direction, Nature Microbiology 9, 1271 (2024). 10.1038/s41564-024-01674-1 PMC11087270 38632342

[58] Yuan J., Fahrner K. A., Turner L., and Berg H. C., Asymmetry in the clockwise and counterclockwise rotation of the bacterial flagellar motor, Proceedings of the National Academy of Sciences 107, 12846 (2010). 10.1073/pnas.1007333107 PMC2919929 20615986

[59] van Albada S. B., Tănase-Nicola S., and ten Wolde P. R., The switching dynamics of the bacterial flagellar motor, Molecular Systems Biology 5, 316 (2009). 19888211 10.1038/msb.2009.74 PMC2779088

[60] Mora T., Yu H., and Wingreen N. S., Modeling Torque Versus Speed, Shot Noise, and Rotational Diffusion of the Bacterial Flagellar Motor, Physical Review Letters 103, 248102 (2009). 20366231 10.1103/PhysRevLett.103.248102 PMC2874687

[61] Meacci G. and Tu Y., Dynamics of the bacterial flagellar motor with multiple stators, Proceedings of the National Academy of Sciences 106, 3746 (2009). 10.1073/pnas.0809929106 PMC2656151 19234112

[62] Meacci G., Lan G., and Tu Y., Dynamics of the Bacterial Flagellar Motor: The Effects of Stator Compliance, Back Steps, Temperature, and Rotational Asymmetry, Biophysical Journal 100, 1986 (2011). 21504735 10.1016/j.bpj.2011.02.045 PMC3077703

[63] Tu Y. and Cao Y., Design principles and optimal performance for molecular motors under realistic constraints, Physical Review E 97, 022403 (2018). 29548155 10.1103/PhysRevE.97.022403 PMC6023414

[64] Cao Y., Li T., and Tu Y., Modeling Bacterial Flagellar Motor With New Structure Information: Rotational Dynamics of Two Interacting Protein Nano-Rings, Frontiers in Microbiology 13 (2022). 10.3389/fmicb.2022.866141 PMC9175137 35694287

[65] Delalez N. J., Wadhams G. H., Rosser G., Xue Q., Brown M. T., Dobbie I. M., Berry R. M., Leake M. C., and Armitage J. P., Signal-dependent turnover of the bacterial flagellar switch protein FliM, Proceedings of the National Academy of Sciences 107, 11347 (2010). 10.1073/pnas.1000284107 PMC2895113 20498085

[66] Yuan J., Branch R. W., Hosu B. G., and Berg H. C., Adaptation at the output of the chemotaxis signalling pathway, Nature 484, 233 (2012). 22498629 10.1038/nature10964 PMC3335734

[67] Lele P. P., Branch R. W., Nathan V. S. J., and Berg H. C., Mechanism for adaptive remodeling of the bacterial flagellar switch, Proceedings of the National Academy of Sciences 109, 20018 (2012). 10.1073/pnas.1212327109 PMC3523824 23169659

[68] Delalez N. J., Berry R. M., and Armitage J. P., Stoichiometry and Turnover of the Bacterial Flagellar Switch Protein FliN, mBio 5, 10.1128/mbio.01216 (2014). PMC4161238 24987089

[69] Branch R. W., Sayegh M. N., Shen C., Nathan V. S. J., and Berg H. C., Adaptive Remodelling by FliN in the Bacterial Rotary Motor, Journal of Molecular Biology 426, 3314 (2014). 25046382 10.1016/j.jmb.2014.07.009 PMC4150818

[70] Antani J. D., Gupta R., Lee A. H., Rhee K. Y., Manson M. D., and Lele P. P., Mechanosensitive recruitment of stator units promotes binding of the response regulator CheY-P to the flagellar motor, Nature Communications 12, 5442 (2021). 10.1038/s41467-021-25774-2 PMC8440544 34521846

[71] Gross S. P., Hither and yon: a review of bi-directional microtubule-based transport, Physical Biology 1, R1 (2004). 16204815 10.1088/1478-3967/1/2/R01

[72] Welte M. A., Bidirectional Transport along Microtubules, Current Biology 14, R525 (2004). 15242636 10.1016/j.cub.2004.06.045

[73] Hancock W. O., Bidirectional cargo transport: moving beyond tug of war, Nature Reviews Molecular Cell Biology 15, 615 (2014). 25118718 10.1038/nrm3853 PMC5014371

[74] Müller M. J. I., Klumpp S., and Lipowsky R., Tug-of-war as a cooperative mechanism for bidirectional cargo transport by molecular motors, Proceedings of the National Academy of Sciences 105, 4609 (2008). 10.1073/pnas.0706825105 PMC2290779 18347340

[75] Soppina V., Rai A. K., Ramaiya A. J., Barak P., and Mallik R., Tug-of-war between dissimilar teams of microtubule motors regulates transport and fission of endosomes, Proceedings of the National Academy of Sciences 106, 19381 (2009). 10.1073/pnas.0906524106 PMC2770008 19864630

[76] Müller M. J. I., Klumpp S., and Lipowsky R., Bidirectional Transport by Molecular Motors: Enhanced Processivity and Response to External Forces, Biophysical Journal 98, 2610 (2010). 20513405 10.1016/j.bpj.2010.02.037 PMC2877362

[77] Kunwar A., Tripathy S. K., Xu J., Mattson M. K., Anand P., Sigua R., Vershinin M., McKenney R. J., Yu C. C., Mogilner A., and Gross S. P., Mechanical stochastic tug-of-war models cannot explain bidirectional lipid-droplet transport, Proceedings of the National Academy of Sciences 108, 18960 (2011). 10.1073/pnas.1107841108 PMC3223464 22084076

[78] D’Souza A. I., Grover R., Monzon G. A., Santen L., and Diez S., Vesicles driven by dynein and kinesin exhibit directional reversals without regulators, Nature Communications 14, 7532 (2023). 10.1038/s41467-023-42605-8 PMC10662051 37985763

